# Diet-induced leukocyte telomere shortening in a baboon model for early stage atherosclerosis

**DOI:** 10.1038/s41598-019-55348-8

**Published:** 2019-12-12

**Authors:** Genesio M. Karere, Michael C. Mahaney, Deborah E. Newman, Angelica M. Riojas, Clint Christensen, Shifra Birnbaum, John L. VandeBerg, Laura Cox

**Affiliations:** 1 Department of Internal Medicine, Wake Forest School of Medicine, Winston-Salem, North Carolina USA; 2 South Texas Diabetes and Obesity Institute and Department of Human Genetics, University of Texas Rio Grande Valley School of Medicine, Brownsville, Texas USA; 3 Southwest National Primate Research Center, Texas Biomedical Research Institute, San Antonio, Texas USA; 4 Department of Genetics, Texas Biomedical Research Institute, San Antonio, Texas USA

**Keywords:** Eukaryote, Telomeres, Predictive markers, Heart failure, Risk factors

## Abstract

Reported associations between leukocyte telomere length (LTL) attrition, diet and cardiovascular disease (CVD) are inconsistent. This study explores effects of prolonged exposure to a high cholesterol high fat (HCHF) diet on LTL in a baboon model of atherosclerosis. We measured LTL by qPCR in pedigreed baboons fed a chow (n = 105) or HCHF (n = 106) diet for 2 years, tested for effects of diet on LTL, and association between CVD risk factors and atherosclerotic lesions with LTL. Though not different at baseline, after 2 years median LTL is shorter in HCHF fed baboons (P < 0.0001). Diet predicts sex- and age-adjusted LTL and LTL attrition (P = 0.0009 and 0.0156, respectively). Serum concentrations of CVD biomarkers are associated with LTL at the 2-year endpoint and LTL accounts approximately 6% of the variance in aortic lesions (P = 0.04). Although heritable at baseline (h^2^ = 0.27, P = 0.027) and after 2 years (h^2^ = 0.46, P = 0.0038), baseline LTL does not predict lesion extent after 2 years. Atherogenic diet influences LTL, and LTL is a potential biomarker for early atherosclerosis. Prolonged exposure to an atherogenic diet decreases LTL and increases LTL attrition, and shortened LTL is associated with early-stage atherosclerosis in pedigreed baboons.

## Introduction

Telomeres are non-coding DNA sequence repeats at the ends of eukaryotic chromosomes (e.g., TTAGGG in vertebrates); they are involved in maintaining genetic stability and integrity by providing protection from damage and fusion. In dividing somatic cells, telomeric regions, like other chromosomal regions, are replicated by DNA polymerase. However, DNA polymerase cannot fully replicate the 3′ end of linear DNA molecules, resulting in progressive shortening of telomeres with repeated cell division. In vertebrates, the telomerase enzyme complex adds DNA to the ends of chromosomes, but is typically only active in certain types of cells, including stem cells, germline cells, granulosa cells, early embryos^1^. In differentiated cells, telomere attrition typically leads to senescence or programmed cell death when mean telomere length reaches a critical value^2,3^. Over the past 2 decades, recognition of telomere dynamics and attrition as fundamental features of cellular senescence has motivated extensive research into their causal roles and potential utility as biomarkers and therapeutic targets for specific cancers^4^.

A large number of epidemiological reports also implicate shorter telomeres as risk factors for many age-related pathologies, including but not limited to components of metabolic disease – i.e., type-2 diabetes^5^, obesity^6^, and cardiovascular disease (CVD)^7^. More specifically, shorter telomere length has been associated with coronary artery disease, as well as atherosclerosis, its consequent complications, disease endpoints, e.g., stroke, and subsequent mortality^8–14^.

The work reported in this paper focuses on the relationship between telomere length and atherosclerosis. A number of mechanisms have been advance to explain the observed associations with atherosclerosis. An oversimplification would organize them into two very general categories. In one, critically shortened telomeres, regardless of when or how established, accelerate disease progression and development of more widely disseminated, later-stage atherosclerosis, as well as consequent CVD through effects on numbers and function of, for example, endothelial progenitor cells needed to effect vascular repair and plaque stabilization^7^. The other explains the correlation between telomere shortening and atherosclerosis as just that: correlated responses to shared exposures to environmental factors that increase systemic inflammation, oxidative stress and cellular aging. Research results consistent with each of these hypotheses may be found readily in the epidemiological literature.

While some studies in human cohorts and families provide evidence that a biologically significant proportion of the inter-individual variance in mean telomere length is attributable to genetic effects^8–10^, there also is evidence that environmental factors, including lifestyle variables which are known to affect atherosclerosis risk, can affect leukocyte telomere length (LTL) as well^11^. Many of these effects on both LTL and atherosclerosis are mediated or hypothesized to be mediated, by, systemic oxidative stress and inflammation^12^. In that case, shortened telomeres, are potentially useful as biomarkers for the cumulative burden imposed by oxidative stress and inflammation on the vasculature – a burden which may result in accelerated atherosclerosis. Key among lifestyle factors that are known to influence systemic inflammation and oxidative stress is diet composition^13^. While the results of all studies are not in complete concordance, a few cross-sectional human studies suggest that some putative atherogenic dietary components, particularly saturated fats, as well as cholesterol and other dietary risk factors for cardiometabolic disease, also may contribute to LTL attrition^15–17^.

Previously, we have shown that prolonged (2-year) exposure to a diet high in cholesterol and fat increases circulating concentrations and/or activity of intrinsic atherosclerosis risk factors, and reliably induces early-stage atherosclerosis in pedigreed baboons from a single, large, six-generation pedigree, which has been extensively characterized at multiple levels of biological organization^14,18^. Here, we take advantage of biomaterials obtained from those same baboons during that study to test the hypothesis that exposure to that atherogenic diet decreases LTL and increases LTL attrition independent of the effects of age and sex. We also test for associations of LTL and LTL attrition with circulating atherosclerosis risk factors previously found to predict extent of atherosclerotic lesions in these animals, as well with the extent of lesions themselves.

## Materials and Methods

### Study subjects and treatment

We used blood samples drawn from live baboons for DNA isolation. All health care, maintenance, and research procedures involving the baboons (*Papio hamadryas*) in this study were managed by the veterinary resources staff of the Southwest National Primate Research Center (SNPRC), Texas Biomedical Research Institute (Texas Biomed), which is accredited by the Association for Assessment and Accreditation of Laboratory Animal Care International. All research procedures were reviewed and approved by the Institutional Animal Care and Use Committee at Texas Biomed. All experiments were performed in accordance with relevant guidelines and regulations.

Baboons from which data were collected for this study were members of a large, six-generation pedigreed breeding colony developed and maintained at SNPRC. The study described in this paper utilizes data from 2 groups of baboons distinguished by diet. Baboons in the control group were fed a baseline diet (chow), low in cholesterol (0.021 mg/kcal) and fat (10% kcal), throughout the study. Baboons in the experimental diet group were fed the chow diet prior to beginning a 2-year dietary challenge during which they were fed a diet high in cholesterol (approximately 1.865 mg/kcal) and fat (40% kcal; HCHF). The diets used in this study have been described in more detail elsewhere^19^. All baboons in this study were fed ad libitum. In summary, the control group was fed chow (LCLF) diet for 2 years while the experimental group was fed HCHF diet for the same duration.

The mean age of animals in diet challenged group (n = 106; 46 females, 60 males) was 10.8 years, with ages ranging from approximately 6 to 17 years. For the control group, we selected age-sex matched adult baboons (n = 106; 47 females, 58 males) from the colony; mean age = 10.9 years and an age range from approximately 6 to 17 years.

### Blood collection

Blood samples (10 ml) were collected through femoral artery venipuncture into EDTA-containing tubes and processed using standard procedures; buffy coats (leukocytes) were stored at −80 °C. For the experimental diet group, samples were collected and processed at 3 time-points: 1–2 weeks prior to the beginning of the 2-year HCHF diet challenge (while on chow), 7 weeks, and at the end of 2-year period during which they were fed the HCHF diet. For the control group, samples were collected at 2 time-points inter-spaced by 2 years.

### DNA isolation

We isolated DNA from 100ul of leukocytes using phenol-chloroform method. Briefly, we mixed cells with lysis buffer and proteinase K and incubated overnight at 55 °C. We transferred lysate to a Phase Lock Gel (PLG) tube (Eppendorf) and centrifuged at 1,500 × g for 2 min at room temperature. Organic extraction was performed twice using phenol, and then with an equal volume of chloroform:isoamyl alcohol (24:1). After the mixture was centrifuged at 1,500 × g for 6 min, the aqueous layer was transferred to a 2 ml tube. To precipitate DNA, we mixed the aqueous layer with 1 volume of 3 M NaOAc and 2.5 volumes of cold 100% ethanol, and inverted the tube 6–8 times. The precipitated DNA was transferred to a new tube containing 70% ethanol, centrifuged for 10 min at 10,000 rpm, and then ethanol was aspirated. The DNA pellet was air-dried, re-suspended in TE buffer (10 mM Tris, 0.1 mM EDTA, pH 7.5) and stored at −80 °C.

### QPCR multiplexing

Relative telomere length was determined using Bio-Rad MyiQ Single Color Real-Time PCR Detection System. DNA samples from diet challenged and control groups for time-points 0 and 2 years were multiplexed side by side in the same 96-well plate to measure the amount of telomere repeats and of a single copy gene (baboon Endothelial lipase; LIPG). Each PCR reaction (20ul) contained 25 ng of DNA, 1X Platinum SYBR qPCR SuperMix-UDG with ROX (MM) and 200 nM of each primer pair for telomere and LIPG. Telomere primer sequences were telg (ACACTAAGGTTTGGGTTTGGGTTTGGGTTTGGGTTAG.TGT) and talc (TGTTAGGTATCCCTATCCCTATCCCTATCCCTATCCCTAACA). LIPG primer sequences were forward (CGGCGGCGGGCGGCGCGGGCTGGGCGGCACTGACTCCAATCGCTTCA) and reverse (GCCCGGCCCGCCGCGCCCGTCCCGCCGATTACAACGGTTCTTGCGGC). The telomere primers were designed such that the telomere amplicon melted at a lower temperature than the LIPG amplicon, and generate a single fixed-length PCR product (79 bp) as described^20^. In addition, each plate contained 81-fold serial dilutions comprising five concentrations of a reference (standard) DNA that ranged from 2.22 to 180 ng together with 1X MM and 200 nM of either telomere or LIPG primer pair for generation of telomere and LIPG standard curves.

All reactions were performed in triplicate, and the cycling profile was Stage 1: 95 °C for 15 min; Stage 2: 2 cycles at 94 °C for 15 s, 49 °C for 15 s; and Stage 3: 32 cycles at 94 °C for 15 s, 62 °C for 10 s, 74 °C for 15 s, 84 °C for 10 s and 88 °C for 15 s. Signals for Telomere repeat Ct values were acquired at 74 °C, and 88 °C for LIPG Ct values.

### Measurement of telomere length

**Figure 1:**
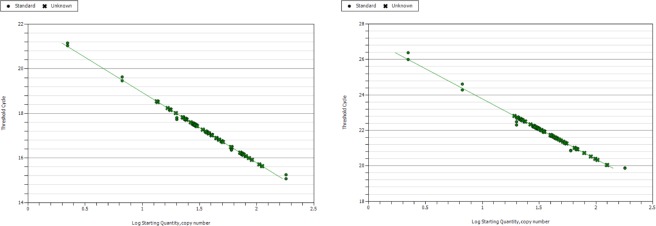
Adjusted standard curves for measurements of (left) baboon leukocyte telomere length, (right) single copy gene (baboon *endothelial lipase*, *LIPG*).

After thermal cycling, we used MyiQ software (Bio-Rad iQ5 2.0 Standard Edition Optical System Software) to generate 2 adjusted standard curves per plate from which telomere length and LIPG copy number (Fig. 1a,b). The MyiQ software generated Ct values matching the DNA content of telomere repeats and LIPG for each of the five standard DNA concentrations. For experimental samples, we used BioRad CFX Manager software together with standard curves to estimate the amount of the standard DNA that matched the experimental sample for the copy number of the telomere (T) and LIPG copy number (S) per sample. We report the mean telomere length of experimental sample/Telomere length of reference sample (T/S) ratio, which is proportional to the mean telomere length per sample.

### CVD related biomarkers

In this study we used data on 13 circulating risk factors which we earlier had reported were significantly correlated with extent of atherosclerotic lesions following the 2-year HCHF diet challenge. Assay methods are described in detail elsewhere^21^. Data obtained from samples collected at baseline and baseline plus 7 weeks into the challenge were analyzed.

### Atherosclerotic lesion assessments

The approach used to assess lesion development in 3 major arteries, the aortic arch, thoracic section of the descending aorta, and the common iliac artery has been described in detail elsewhere^21,22^. Briefly, baboons were humanely euthanized after 2 years on the HCHF diet and the 3 arterial sections were harvested in the course of standard necropsy procedures. Following dissection, preparation, and application of a lipophilic stain, lesions (fatty streaks and/or raised fibrous plaques) were visually identified and photographed, and the photographs were imported into an image analysis system and percent area covered by lesions was quantified as previously described^23^. Note: Raised lesions most resembled AHA lesion Types Va or Vc^24^.

### Analytical methods

Summaries of raw data are presented by cohort, time-point, and sex as means, medians, standard deviations, and ranges. The data are in a sense derived “opportunistically” for this particular study. That is, the study is not one in which cases and controls could be selected based on baseline LTL and matched for sex and age. Further, normality of distributions could not be guaranteed. As some departures from univariate normality (e.g., skew) affect the mean more than the median, we instead use the latter as the measure of central tendency in our *initial* comparisons of raw data. We employ a Wilcoxon-Mann-Whitney U test of medians that implements an exact permutation approach which is robust to the presence of outliers^25^.

Further, because the data come from related animals (from the large, six-generation pedigree alluded to earlier), assumptions of independence of observations on which many statistical tests rely also cannot be guaranteed (note: mean kinship coefficient between all pairs of animals within each of the two cohorts is approximately 0.14, and for the combined cohort, 0.17 – i.e., between half and full siblings). To address this potential bias, in all remaining analyses we utilize a maximum likelihood-based variance decomposition approach (SOLAR^26^) which accounts for kinship in data from pedigrees of arbitrary size and complexity. To address possible departures from multivariate normality, data analyzed using this approach are i-normalized quantile scores (i.e., inverse Gaussian normalization), the distribution of which are symmetric about the mean and median. We use this approach to decompose the phenotypic covariance among related animals into genetic and environmental components and then model the phenotype of an individual as a general linear function of the trait, its mean, covariates and their regression coefficients, plus additive genetic values and non-genetic deviations. Here we test for effects of covariates on the phenotype by comparing the maximum likelihood of a model in which the mean effect is estimated to that of a model where that covariate’s effect is constrained to equal zero (the null model).

## Results

### Descriptive statistics and initial impressions

*Baseline*. Initial examination and analyses of raw (untransformed) data (Table 1) shows the 2 cohorts to be well matched on LTL prior to the 2-year diet challenge when both were consuming the LCLF diet. Median LTL in the 2 cohorts is not different (sexes combined: P = 0.8204; females: P = 0.5548; males: P = 0.4256). *Between cohorts*. For both cohorts, the within-cohort comparisons of the sexes indicate that the median LTL in female baboons is greater than that in males (control: P = 0.0094; challenge: P = 0.0076).

**Table 1:** Telomere-to-Single Copy Gene (T/S) Ratios in Pedigreed Baboons: Descriptive Statistics.

T/S Ratio						
*Control Cohort*						
	*Baseline (chow diet)*			*Baseline* + *2 Years (chow diet)*		
	Females	Males	Total	Females	Males	Total
Mean	1.07	0.97	1.01	0.97	0.91	0.94
Median	0.96	0.87	0.91	0.91	0.89	0.90
SD	0.27	0.23	0.25	0.21	0.12	0.17
Minimum	0.78	0.73	0.73	0.77	0.74	0.74
Maximum	1.59	1.61	1.61	1.75	1.56	1.75
Range	0.81	0.88	0.88	0.98	0.82	0.91
***Experimental Cohort***						
Mean	0.97	0.91	0.94	0.89	0.86	0.87
Median	0.97	0.90	0.92	0.89	0.84	0.86
SD	0.13	0.09	0.12	0.08	0.08	0.08
Minimum	0.78	0.77	0.77	0.74	0.74	0.74
Maximum	1.47	1.31	1.47	1.15	1.16	1.16
Range	0.69	0.54	0.70	0.41	0.42	0.42

#### Baseline plus 2 years

When compared to those at baseline, observations made at time point 2 suggest an effect of the HCHF diet. The difference between median LTL at the 2 time points was not significant in the control cohort, which had consumed the LCLF diet for 2 years (combined sexes: P = 0.4057; females: P = 0.1042; males: P = 0.7220); but it was in the cohort that had consumed the atherogenic HCHF diet (combined sexes: P = 0.0000007; females: P = 0.0076; males: P = 0.0008). Median LTL in the control cohort was longer than that in the experimental cohort (sexes combined: P = 0.0005).

At this time point, the results of within-cohort comparisons of the sexes are similar to those at baseline. However, while median LTL in females is absolutely greater than that in males in both cohorts, that difference is statistically significant only in the experimental cohort (control: P = 0.2132; experimental: P = 0.0069).

### Sex and age effects on inter-individual variation in LTL: Separate cohorts

We maximized a model with sex and age terms on the i-normalized LTL data for each of the cohorts separately at each time point to estimate and test the significance of mean effects that might underly the observations above.

#### Baseline

Sex, age, and an age-by-sex interaction all exerted significant mean effects on LTL in the control cohort (P = 0.004; P = 0.00002; and 0.026, respectively). In the experimental cohort, the mean effects of sex and age were significant (P = 0.028; P = 0.025) but no age-by-sex interaction was detected. In both cohorts the signs of the mean effects were as expected: sex (female) and age were, respectively, positively and negatively correlated with LTL.

#### Baseline plus 2 years

For the control group, at time point 2, only the mean effects of an age-by-sex interaction are statistically significant (P = 0.031), the net effect of which may underlie that apparent decrease in difference between LTL in females and males (Table 1). While the signs are in the expected orientations – i.e., sex is positively correlated (P = 0.113) with LTL and age is negatively correlated (P = 0.232] – the magnitudes of each are not large. Analyses of time point 2 data for experimental animals reveals a small, suggestively significant sex effect (P = 0.056) and no age effect (P = 0.266).

### Diet, sex and age effects on inter-individual variation in LTL: Combined cohorts

#### Baseline

We maximized a quantitative genetic model for LTL on data from both cohorts at baseline when all animals had been fed the LCLF diet. In addition to the additive genetic and effects of unmeasured environmental and non-additive genetic factors, this model contained sex and age as covariates. Likelihood ratio tests showed that each exerted a significant mean effect on LTL (P_sex_ = 0.000304 and P_age_ = 0.0273) and the effects of each exhibited the expected directionality reported in the literature and seen in our raw data: i.e., mean LTL is greater in female baboons than in males and smaller in older individuals (Table 2). Also, the estimated proportion of the residual phenotypic variance in LTL attributable to the effects of genes, the heritability, was significant, but modest (h^2^ = 0.27 ± 0.19, P = 0.027).

**Table 2:** Mean effects of sex, age, and diet and additive effects of genes on LTL in pedigreed baboons at two timepoints: Cohorts combined.

Baseline			Baseline + 2 years	
Parameter	MLE ± S.e.	P	MLE ± s.e.	P
βsex	0.516 ± 0.141	0.0003	0.218 ± 0.169	0.2975
βage	−0.089 ± 0.040	0.0274	−0.048 ± 0.031	0.1976
βdiet	N/A	N/A	−0.486 ± 0.143	0.0009
h^2^	0.270 ± 0.128	0.0266	0.457 ± 0.242	0.0038

Baseline: diet is LCLF for both; Baseline + 2 years: Control group fed LCLF diet and experimental group fed HCHF atherogenic challenge diet for 2 years.

MLE ± s.e.: maximum likelihood estimates of parameters and their standard errors.

P: Probability a parameter estimate equals zero (by likelihood ratio test).

Sex: A dichotomous (0, 1) variable. Estimate of mean effect of being female.

Age: Continuous variable (decimal years).

Diet: Dichotomous variable (0, 1). Estimate of mean effect of HCHF diet.

h^2^: Heritability or proportion of residual phenotypic variance in the phenotype due to the additive effects of genes.

#### Baseline plus 2 years

We applied the same approach to sex- and age-adjusted residuals of data collected from both cohorts after the control and experimental cohorts had been fed the LCLF and HCHF diets, respectively, for 2 years. The estimated mean effect of diet, independent of age and sex, was significant (P = 0.000857); and, consistent with our earlier observations, LTL was smaller in baboons who had been fed HCHF diet for 2 years. The estimated heritability also was significant (h^2^ = 0.46 ± 0.24).

### Diet effects on change in LTL

As should be expected from the results of our comparisons of median LTL in the 2 cohorts at time point 2 (above), change in LTL (ΔLTL), or telomere attrition itself, is greater in animals who consumed the HCHF diet for 2 years than in the controls who ate the LCLF diet during the same interval (P = 0.006). These analyses of the raw data disclose no significant between-sex difference in ΔLTL within either of the cohorts (control: P = 0.3681; experimental: P = 0.1554).

We used the approach described above in 2 series of analyses of ΔLTL: one with data from the separate cohorts and the other with data from the cohorts combined. The first series of ΔLTL was calculated as the difference between the i-normalized in raw LTL from the individual cohorts and the second from the cohorts combined. 1) Separate cohorts. Our analysis of the control cohort data finds a significant mean positive effect of age in the control cohort (P = 0.003); that is, ΔLTL was greater in older control baboons. This effect accounts for approximately 20% of the variance in ΔLTL in that cohort. Analysis of data from the experimental cohort finds no significant evidence for a significant mean effect of sex or age on ΔLTL. 2) Combined cohorts. To test for an effect of diet on change in LTL within this same framework, we calculated ΔLTL as the difference between the i-normalized sex- and age-adjusted residuals for LTL at the 2 time points. As inferred from comparisons of median ΔLTL in the preceding analyses (above), the mean effect of diet on ΔLTL is positive; i.e., ΔLTL is greater in animals fed the HCHF diet for approximately 2-years (P = 0.0156). We observed no evidence of a significant additive genetic effect (heritability) on ΔLTL in either of the cohorts.

### Biomarkers of cardiovascular disease risk and LTL

Results from analyses of well-recognized CVD risk factors, 13 circulating biomarkers of lipoprotein metabolism, oxidative stress, and inflammation assayed in samples collected at 3 time-points, i.e., time-point 0, 0 + 7 weeks, and 0 + 2 years are presented in Table 3. None of the serum biomarkers assayed in the time-point 0 samples are correlated with LTL at 2 years; however, 4 of them, quantified in samples obtained at 7 weeks, are correlated with LTL at 2 years. Sex and age adjusted concentrations of very low plus low density lipoprotein cholesterol (V + LDLC) and apolipoprotein E (apo E) are negatively correlated with and account for approximately 5.8% to 4.6% of the variance in LTL following the 2-year challenge (P = 0.015 and P = 0.032). The mean effects of the activity of paraoxonase (PON1], an inhibitor of high density lipoprotein oxidation, and adjusted total antioxidant status (TAS], a measure of peroxyl-scavenging capacity^27^, on mean LTL are positive (P = 0.035 and P = 0.025, respectively]. On average, they are correlated with longer LTL. PON1 and TAS biomarkers each account for approximately 5% of the variance in LTL. There is no evidence for any suggestively significant (0.5 ≤ P ≤ 0.10] effects of any other of the 13 CVD-related biomarkers analyzed in this study.

**Table 3:** Correlations: LTL and biomarkers of lipid metabolism, inflammation and oxidative stress by time on HCHF diet.

Trait	Baseline			Baseline + 7 weeks			Baseline + 2 years		
	R^2^	r	P(r = 0)	R^2^	r	P(r = 0)	R^2^	r	P(r = 0)
HDLC	0.0164	0.090	0.373	0.008	0.089	0.378	0.010	0.102	0.313
V + LDLC	0.0260	−0.160	0.112	0.058	−0.241	0.015	0.005	−0.069	0.498
TG	0.0020	−0.040	0.693	0.016	0.126	0.212	0.002	−0.048	0.635
apo A1	0.0037	0.192	0.055	0.002	0.045	0.657	0.000	0.003	0.976
apo B	0.0049	0.070	0.488	0.004	0.020	0.843	0.001	0.030	0.767
apo E	0.00008	0.009	0.929	0.046	−0.214	0.032	0.002	−0.046	0.649
CRP	0.0002	0.013	0.897	0.015	0.122	0.226	0.004	−0.062	0.540
oxLDL	0.0044	−0.066	0.514	0.012	0.110	0.272	0.0003	0.018	0.859
IL8	0.0060	−0.077	0.446	0.002	0.045	0.667	0.010	−0.101	0.317
LpPLA_2_	0.0034	0.058	0.567	0.012	0.110	0.276	0.004	−0.061	0.547
PON1	0.0163	0.127	0.207	0.045	0.212	0.035	0.001	0.038	0.707
TAS	0.0106	0.103	0.378	0.050	0.224	0.025	0.007	0.085	0.400
VWF	0.0005	0.023	0.820	0.014	0.118	0.242	0.001	0.027	0.790

Abbreviations: For traits, TSC: total serum cholesterol, HDLC: high density lipoprotein cholesterol, V + LDLC: very low + low density lipoprotein cholesterol (V + LDLC), TG: triglycerides, apo A1: apolipoprotein A1, apo B: apolipoprotein B, apo E: apolipoprotein E, CRP: C-reactive protein, IL8: interleukin 8, LpPLA_2_: lipoprotein associated phospholipase 2 activity, PON1: paraoxonase 1 activity, TAS: total antioxidant status, and VWF: von Willebrand factor. For table column headers, R^2^: proportion of the variance in LTL attributable to the biomarker; correlation between the biomarker and LTL; P(r = 0): probability that the correlation between the biomarker and LTL equals zero, which is equal the probability that mean effect of the biomarker (β) on LTL equals zero in the model.

### Association of LTL and LTL attrition with extent of atherosclerotic lesions

We find significant evidence that LTL is associated with the extent of atherosclerotic lesions quantified as the percent area in a section of one of the three arteries harvested during necropsy from baboons following the 2-year atherogenic diet challenge: the descending aorta. LTL accounted for approximately 6% (P = 0.010) of the variance in lesion extent in that vessel. The relationship is negative (r = −0.247), with smaller ratios being associated with greater proportions of arterial area covered by an atherosclerotic lesion (Table 4). We detect a similarly small but significant association between the magnitude of LTL attrition (ΔT/S ratio) and the extent of atherosclerotic lesions in the common iliac artery. ΔLTL accounts for approximately 4% (P = 0.036) of the variance in lesion extent in that vessel (Table 5). However, LTL at baseline is not predictive of extent of atherosclerotic lesions in any of the three arteries (for aortic arch, P = 0.89; common iliac artery, P = 0.20; and descending aorta, P = 0.28) in the current study.

**Table 4:** Correlations between LTL and extent of atherosclerotic lesions at three sites.

Trait	R^2^	r	P(r = 0)
Aortic arch	0.0157	−0.125	0.201
Common iliac artery	0.0027	−0.052	0.596
Descending aorta	0.0609	−0.247	0.010
Sum of lesion extent at three sites	0.0219	−0.148	0.130
Mean lesion extent at three sites	0.0220	−0.148	0.130

R^2^: proportion of the variance in lesion extent attributable to LTL; correlation between LTL and lesion extent; P(r = 0): probability that the correlation between LTL on extent of lesion (r) equals zero, which is equivalent to the probability that the mean effect LTL (β) on lesion extent equals zero in the model.

**Table 5:** Correlations between ΔLTL and extent of atherosclerotic lesions at three sites.

Trait	R^2^	r	P(r = 0)
Aortic arch	0.0034	−0.059	0.595
Common iliac artery	0.0408	−0.202	0.036
Descending aorta	0.0190	−0.137	0.170
Sum of lesion extent at three sites	0.0114	−0.106	0.279
Mean lesion extent at three sites	0.0114	−0.109	0.266

R^2^: proportion of the variance in lesion extent attributable to ΔLTL; correlation between ΔLTL and lesion extent; P(r = 0): probability that the correlation between ΔLTL on extent of lesion (r) equals zero, which is equivalent to the probability that the mean effect of ΔLTL (β) on lesion extent equals zero in the model.

## Discussion

In the last few decades, major risk factors for atherosclerosis have been identified and many novel intrinsic risk factors continue to be nominated. For any of these, finding evidence to support an association with vascular pathology and to implicate a mechanism by which their effects are manifested will be a key step to assessing the likelihood that further study ultimately could result in the development of diagnostic, preventive, therapeutic, or prognostic approaches. Other key steps will be elucidating interactions between some of these novel intrinsic risk factors and modifiable extrinsic environmental factors already known to increase risk for the disease. High fat diet is one of the major traditional environmental risk factors for atherosclerosis and one relatively novel intrinsic risk factor for atherosclerosis is telomere length^15^.

Based on our study of 2 cohorts of pedigreed baboons, which for 2 years consumed either a control diet, low in cholesterol and fat, or an experimental HCHF diet, known to be atherogenic, we derive 4 inferences. The 2 most salient of these are that 1) prolonged consumption of the HCHF diet leads to significantly lower median LTL, independent of the effects of aging and 2) shorter LTL and the decrease in LTL (ΔLTL) following the diet challenge are associated with the extent of early atherogenic lesions. Additionally, we infer that 3) variation in inflammation and oxidative stress contribute to variation in LTL shortening and 4) the additive effects of genes account for a significant proportion of observed variation in LTL before and after prolonged consumption of the HCHF diet.

The central finding of this study is that prolonged exposure to the atherogenic HCHF diet results in significant LTL attrition. Rodent models have previously demonstrated a similar finding, however this is the first study to show the relationship between HCHF diets and LTL attrition in primates^11^. While consistent with expectations based on epidemiological studies of human populations and cohorts in which consumption of diets that vary with respect to the relative proportions of dietary fat (particularly saturated fats and polyunsaturated fats) is correlated with variation in LTL^11,13,16,17,28^, we interpret the results of our study to be indicative of a causal relationship. Support for this inference derives from 2 principal observations: 1) decreased LTL is independent of age and sex effects in the challenge animals and 2) there is no significant change in LTL over the same period in the control animals. The effect of the 2-year exposure to the HCHF diet on LTL is substantial. As much as 40% of the phenotypic variance in LTL is due to the effects of the HCHF diet.

There is general agreement that inflammation and oxidative stress contribute to LTL attrition^29^ and the HCHF diet used in this study certainly is pro-inflammatory. Associations between LTL at 2 years and 4 biomarkers of inflammation and/or oxidative stress implicate these processes in the observed diet-induced LTL: i.e., serum concentrations of 2 biomarkers of lipid metabolism, V + LDLC and apo E, and the activities of 2 indicators of resistance to oxidative stress, PON1 and TAS, which, respectively, are negatively and positively correlated with LTL. The effects of these biomarkers are relatively small, with each accounting for 4% to 6% of the variance in LTL at 2 years. The fact that it is the 7-week values for the biomarkers, and not those from time-point 0 or 2 years, for which we detect effects on LTL at 2 years would be consistent with a causal relationship. While the work reported here cannot conclusively demonstrate such a relationship, we note that elevated biomarkers of lipid metabolism, inflammation, and oxidative stress measured at this same time-point (i.e., 7 weeks into the HCHF diet challenge) also were stronger predictors of the extent of atherosclerotic lesions at 2 years in same pedigreed baboons than those assayed at other time-points^19^.

While both LTL and ΔLTL are significantly correlated with the extent of atherosclerotic lesions in 2 major arteries, the effect sizes implicated are small, accounting for between 4% and 6% of the variance in the descending aorta and common iliac artery, respectively. Nonetheless, the signs of the correlations are consistent with expectations: shorter LTL and greater LTL attrition (over the 2-year period) are associated with increased lesion extent.

Based on the results of studies in humans, other researchers have suggested that short LTL at any time may be a more important risk factor for atherosclerosis than LTL attrition. One of these is that inter-individual variation in LTL, which has a significant heritable component, is established by rapid attrition in early life, after which age-related attrition rates do not differ significantly regardless of differences in risk^30^. The other is that short LTL at any time increases susceptibility to atherogenic factors and that LTL attrition is associated with increased severity of atherosclerosis. Our study is not designed as an explicit test or either hypothesis.

Our study design also does not allow us to determine when during the 2-year diet challenge this association is established – i.e., when shorter LTL marks an increase in risk for atherosclerosis. However, an earlier study in which baboons from this same breeding colony consumed the same HCHF diet for only 7 weeks found increased prevalence of senescence in vascular endothelial cells^31^, suggesting that diet-induced LTL attrition, a biomarker of, if not a contributor to, cellular senescence *could* also begin very soon after starting the atherogenic diet.

Again, the focus of our research is *early-stage* atherosclerosis. We are not aware of a previous report of a relationship between LTL and early-stage atherosclerosis in either humans or nonhuman primates. Those human studies to which we alluded earlier in this paper find that short LTL is associated with clinically appreciable indicators of CVD, examples of which include, but are not limited to, angiographically detected severe triple-vessel coronary artery disease^15^; coronary artery calcium^32^; and complicated carotid artery plaques^21,33^; as well as with composite measures of cardiovascular health^34,35^. But those observations reflect well-developed, clinically appreciable, later-stage atherosclerosis in large-scale cross-sectional cohort studies of adults. Citing the results of 2 such studies^36,37^, Riezschel *et al*.^7^ posit that the short LTL-atherosclerosis association does not extend to early-stage disease.

We believe the different picture painted by our results lies in large part in the experimental study design, which enhances our ability to quantify and control and many factors of interest with greater precision than is possible in most epidemiological studies in human populations/cohorts. These include, for example, having confidence in the compositions of both the baseline and experimental diets, which are fully defined and completely uniform throughout the course of the diet challenge; minimization of exposures to extraneous “lifestyle” and other environment factors; accuracy of the additive genetic “background;” and the validity of the data on the presence, size, and nature of atherosclerotic lesions, as they were obtained by direct observation (see earlier publication^21^).

## Summary and Conclusion

We have shown that a diet previously demonstrated to be atherogenic in captive baboons from a pedigreed breeding colony affects LTL and that the effects of diet are in addition to those of aging. We also have shown that diet-induced shorter LTL is negatively associated with extent of vascular lesion development in early-stage atherosclerosis. Both observations have been made in the same individuals, in the course of the same study.

To our knowledge this is the first prospective, longitudinal, experimental study of its kind in a primate species. Although highly informative, such study designs are impractical, if not impossible, in humans as they require accurate knowledge of and control of composition and consumption of a diet known to reliably induce the disease state of interest, the ability to control for background genetic variation in order to maximize the signal-to-noise ratio, and the ability to accurately assess the pre-clinical disease state. But as we have shown here, in the case of early-stage atherosclerosis, all of these issues can be addressed so that such studies can be conducted successfully with a relevant animal model for early-stage atherosclerosis: the pedigreed baboon. Given its phylogenetic proximity and consequent genetic, physiological, and anatomical similarity to our own species, this nonhuman primate model can be used profitably to investigate the role(s) of telomeres and/or telomere attrition in vascular changes accompanying early stages of, or even presaging, atherogenesis in humans.

## Data Availability

For the current study, datasets generated during and/or analyzed are available upon request from the corresponding author on reasonable request.
